# Cerebrospinal fluid markers in incident pediatric-onset multiple sclerosis: a nationwide study

**DOI:** 10.1038/s41598-021-97543-6

**Published:** 2021-09-17

**Authors:** Kyla A. McKay, Ronny Wickström, Jan Hillert, Virginija Danylaite Karrenbauer

**Affiliations:** 1 Department of Clinical Neuroscience, Karolinska Institutet, Stockholm, Sweden; 2 Center for Molecular Medicine (CMM) L8:05, Karolinska University Hospital, 171 76 Stockholm, Sweden; 3 Neuropediatric Unit, Astrid Lindgren’s Childrens’s Hospital, Karolinska University Hospital, Stockholm, Sweden; 4 Theme Neuro, Karolinska University Hospital, Stockholm, Sweden

**Keywords:** Multiple sclerosis, Disability

## Abstract

To investigate whether cerebrospinal fluid (CSF) markers differ between pediatric-onset multiple sclerosis (PoMS, onset < 18 years) and adult-onset (AoMS), and whether these markers are associated with clinical outcomes among PoMS. Prospective nationwide registry study of incident MS, including persons with a CSF sample < 3 years post-MS onset. We compared CSF oligoclonal band (OCB) status, immunoglobulin G (IgG) index levels, and mononuclear cell count between PoMS and AoMS. Within the PoMS cohort we analyzed the association between CSF markers, relapse rate and Expanded Disability Status Scale (EDSS) score, using negative binomial regression and generalized estimating equations, respectively. The cohort consisted of 130 PoMS and 3228 AoMS cases. The PoMS group had higher odds of OCB-positivity (odds ratio: 2.70; 95% CI 1.21–7.67). None of the CSF markers were associated with relapse rate in the PoMS cohort; however, OCB-positivity was associated with higher EDSS scores. This study suggested that PoMS more commonly display CSF evidence for intrathecal IgG production than AoMS. Further, we found evidence of a relationship between OCB-positivity and subsequent disability, suggesting that they could play a role in the prognostication of MS in children.

## Introduction

Pediatric-onset MS (PoMS) has a unique clinical profile compared to the more common adult-onset MS (AoMS), including increased focal inflammation early in the disease, rarity of primary progressive disease course, and slower long-term disability progression from onset^1–3^. Cerebrospinal fluid (CSF) markers have played an important role in establishing an MS diagnosis, and appear to possess some prognostic qualities in the general MS population^4^. Prior work has largely explored PoMS and AoMS separately^4–7^, leaving unanswered questions regarding potential differences in CSF markers between children and adults with MS. Further, there is limited understanding of the potential prognostic utility of CSF markers in PoMS. We aimed to investigate whether routinely-analyzed CSF markers differ between incident cases of PoMS and AoMS, and whether any of these markers are associated with clinical outcomes, including relapse rate and disability progression among the PoMS cohort. Given the prior evidence of pronounced inflammatory patterns on MRI and increased rates of clinical relapses among persons with PoMS (relative to AoMS)^8^, we hypothesized that the PoMS patients would be more likely to exhibit high levels of CSF markers indicative of intrathecal IgG production than the AoMS. Further, we expected that these would be associated with an increased risk of relapse, but not disability, as these markers are indicative of acute inflammation.

## Methods

### Study population

We performed a cohort study using prospectively-collected data from the Swedish MS (SMS) registry, which collates information from all 64 neurology clinics in the country^9^. We included only incident cases of definite MS (CIS were excluded) according to the McDonald criteria^10–12^. The SMSreg is routinely evaluated for accuracy and has been found to be highly accurate in terms of MS diagnoses^13^. Cases must have had a lumbar puncture (LP) within 3 years of their MS onset (first clinical event). Only LPs performed on or after January 1st, 2001 were included, corresponding with the start of the nationwide SMSreg. The date of LP was considered the baseline date. All patients must have had a at least 1 year of follow-up following their baseline visit, and the end date of follow-up was December 31st, 2019.

PoMS was defined as MS onset < 18 years of age, in line with consensus from the International Pediatric MS Study Group^14^. The adult-onset cohort included all persons who met the inclusion criteria and had an onset age of 18 years or older. Due to the rarity of primary progressive MS in the pediatric population, and to ensure comparability between groups, we selected only relapsing-onset patients in both cohorts.

### Study exposures and outcomes

We analyzed CSF oligoclonal band (OCB) status, immunoglobulin G (IgG) index levels, and mononuclear cell count in the full cohort. These outcomes were selected because they are routinely collected in the neurology clinic, and therefore of clinical relevance. Detailed clinical information was collected in the SMSreg prospectively, including information on relapses and neurological disability. Relapses are recorded by the neurologist as new or recurrent neurologic symptoms not associated with fever or infection that lasted for at least 24 h and were accompanied by new neurologic signs. The expanded disability status scale (EDSS)^15^ is performed in the clinic on an approximately annual basis by an MS-specialist neurologist. The scale ranges from 0 (no disability) to 10 (death due to MS)^15^.

### Statistical analysis

Hypotheses were formulated before the registry data extraction. Continuous variables were compared using the student’s t-test, or Wilcoxon-rank sum test. Categorical variables were compared by the chi-squared test or the Fisher’s exact test. OCB status is a binary outcome (positive vs negative), but we also categorized the continuous CSF outcomes as “high” vs “normal” using established normal ranges in Sweden (IgG index normal range ≤ 0.70; mononuclear cell count normal range < 5 × 10^6^/μl). We used logistic regression analysis, with results presented as odds ratios (OR) and 95% confidence intervals (95% CI), adjusted for sex and region of Sweden (North, Central, South).

Within the PoMS cohort, we explored the relationship between CSF markers and relapse rate in the 5 years following the baseline date. We used negative binomial regression, as the relapse rate was an over-dispersed count variable. Covariates that were considered included age at baseline, sex, region of Sweden, relapse status, and disease-modifying therapy (DMT) exposure, with time from baseline included as an offset. DMTs were classified as moderate-efficacy (interferon-beta, glatiramer acetate, dimethyl fumarate, teriflunomide) or high-efficacy (rituximab, ocrelizumab, natalizumab, fingolimod, alemtuzumab, mitoxantrone, and cladribine)^16,17^, and analyzed as time-varying covariates in the 5 years post-baseline. Findings were reported as incident rate ratios (IRRs) with 95% confidence intervals.

EDSS was similarly analyzed in the 5 years following baseline. We used generalized estimating equations, with an exchangeable correlation structure in order to use all EDSS scores recorded during this period for each individual. The EDSS score was modeled as a linear variable, adjusting for age at onset, time from baseline, sex, region of Sweden, and time on first- and second-line DMTs, with results presented as beta-coefficients and standard deviations (SD). Given that adjusting for multiple reduction reduces the type I error for null associations and increases the type II error for those associations that are not null, we did not control for multiple comparisons in this study. Statistical analyses were performed using R: a Language and Environment for Statistical Computing v.4.0.2 (R Foundation for Statistical Computing, Vienna, Austria; 2020).

The study was approved by the Regional Ethical Review Board of Stockholm, and informed consent was provided from patients for the collection of their clinical information. All methods were performed in accordance with the relevant guidelines and regulations, and informed consent was obtained from all participants and/or their legal guardian.

## Results

Between 2001 and 2019, 429 PoMS and 7842 AoMS had a lumbar puncture (LP) recorded in the SMSreg. Among whom, 130 incident cases of PoMS and 3228 AoMS had the LP performed within 3 years of their disease onset. There were no differences between the groups in terms of sex ratio, disease course, DMT or steroid exposure at baseline, nor region of Sweden (Table 1). The PoMS baseline ages ranged from 6 to 17, but the cohort was comprised predominantly of adolescents (≥ 13 years; 93%). Less than 5% of the full cohort (143/3358) were exposed to a DMT at the time of the CSF analysis.

**Table 1:** Clinical and demographic features of the cohort, comparing the pediatric- and adult-onset MS cases.

	Pediatric-onset MS	Adult-onset MS	p-value
n	130	3228	
Onset age (median [IQR])	16.77 [15.28, 17.51]	33.17 [26.62, 41.20]	< 0.001^a^
Age at baseline (median [IQR])	17.31 [15.99, 18.11]	33.83 [27.27, 41.94]	< 0.001^a^
Sex			0.991^b^
Female/male (%)	91/39 (70.0/30.0)	2274/954 (70.4/29.6)	
Region, n (%)			0.24^b^
South	38 (29.2)	954 (29.6)	
Central	67 (51.5)	1817 (56.4)	
North	25 (19.2)	453 (14.1)	
DMT Exposure (in first 5 years of disease), n (%)			0.22^b^
None	13 (10.0)	330 (10.2)	
Moderate-efficacy only	50 (38.5)	1432 (44.4)	
High-efficacy only	36 (27.7)	653 (20.2)	
Switch moderate to high-efficacy	31 (23.8)	813 (25.2)	
DMT exposure prior to baseline, n (%)			0.74^b^
Interferon-beta	4 (3.1)	83 (2.8)	
Rituximab	0 (0.0)	10 (0.3)	
Glatiramer acetate	0 (0.0)	8 (0.3)	
Dimethyl fumarate	0 (0.0)	10 (0.3)	
Natalizumab	1 (0.8)	13 (0.4)	
Other*	0 (0.0)	14 (0.4)	
Relapse status at baseline, n (%)			0.41^b^
On relapse	43 (33.1)	947 (29.3)	
In remission	87 (66.9)	2281 (70.7)	
Steroid treatment at baseline, n (%)	16 (12.3)	370 (11.5)	0.99^b^

*IQR* interquartile range.

^a^By Wilcoxon rank-sum test.

^b^By chi-squared test.

*Other DMTs included teriflunomide, ocrelizumab, fingolimod, alemtuzumab, mitoxantrone, and cladribine.

When exploring crude CSF levels, we found that the pediatric cohort had a higher proportion of OCB-positive individuals, IgG index, and mononuclear cell count compared to the adult-onset cohort, though only OCB status and IgG index were statistically different between groups (Table 2).

**Table 2:** Crude cerebrospinal fluid values compared between pediatric- and adult-onset cases within 1 year of MS onset.

CSF Marker	Pediatric-onset N = 130	Adult-onset N = 3228	p-value
Oligoclonal bands, n (%)	122 (96.1)	2848 (90.0)	0.04^a^
IgG index (median [IQR])	0.98 [0.71, 1.37]	0.86 [0.64, 1.22]	0.03^b^
High IgG index, n (%)	72 (75.0)	1850 (67.0)	0.13^c^
Mononuclear cell count (median [IQR])	6.00 [0.00, 14.00]	4.00 [0.00, 10.00]	0.06^b^
High mononuclear cell count, n (%)	82 (72.6)	2088 (74.1)	0.80^c^

*IQR* interquartile range.

^a^By Fisher’s exact test.

^b^By Wilcoxon rank-sum test.

^c^By chi-squared test.

When adjusted for sex and region of Sweden (relapse status was non-significant and not included in the models), the PoMS group were found to have 2.70 (95% CI 1.21–7.67) times higher odds of detectable OCB bands in their CSF (Table 3). When the continuous outcomes (IgG index and mononuclear cell count) were dichotomized into “normal” or “high” values we found no differences between groups.

**Table 3:** Odds ratios and 95% confidence intervals for the odds of having “positive” or “high” CSF values in the pediatric-onset patients compared to the adult-onset patients (reference group).

CSF marker	Odds ratio (95% CI)	Adjusted^a^ odds ratio (95% CI)
**Oligoclonal bands**		
Positive (vs negative)	2.72 (1.22–7.72)	2.70 (1.21–7.67)
**IgG index**		
High (vs normal)	1.48 (0.93–2.40)	1.46 (0.92–2.38)
**Mononuclear cells**		
High (vs normal)	0.92 (0.61–1.43)	0.94 (0.61–1.49)

^a^Adjusted for sex and region of Sweden.

In the 5 years following baseline (median follow-up time: 5.0 years [interquartile range: 5.0–5.0 years), 48% of PoMS patients experienced no relapses, while 22% had one, and 30% had more than one recorded. None of the CSF markers were associated with subsequent relapse rate (Table 4).

**Table 4:** Relationship between CSF values and relapse rate among pediatric-onset patients in the 5 years following baseline, using negative binomial regression.

CSF marker	IRR (95% CI)	Adjusted IRR (95% CI)^a^
**Oligoclonal bands**		
Present (vs absent)	1.41 (0.36–5.33)	1.61 (0.42–6.12)
**IgG index**		
High (vs normal)	1.28 (0.70–2.35)	1.12 (0.62–2.04)
**Mononuclear cells**		
High (vs normal)	0.71 (0.41–1.22)	0.70 (0.40–1.21)

*IRR* incident rate ratio.

^a^Adjusted for age at sampling, sex, region of Sweden, and time on first- and second-line DMTs.

Of the full cohort, 118 (90.8%) of the PoMS patients had at least one EDSS score recorded within 5 years of baseline and were included in the analysis. The median number of EDSS scores recorded was 5 (IQR: 3–8). At a population level, the cohort had mild disability (median EDSS: 1.0; IQR: 0.0–2.0) during this period. OCB-positivity within the first 3 years of disease was associated with a higher EDSS score, by 0.72 points (SD: 0.27), on average. Neither IgG index level nor mononuclear cell count were associated with subsequent disability levels (Table 5).

**Table 5:** Relationship between CSF values and disability, as measured by the EDSS (continuous), in the 5 years following baseline, using generalized estimating equations.

CSF marker	Β-coefficient (SD) of EDSS	p-value	Adjusted Β-coefficient (SD) of EDSS*	p-value*
Oligoclonal band (positive)	0.68 (0.21)	0.002	0.72 (0.27)	0.001
**IgG index**				
High (vs normal)	0.07 (0.12)	0.55	0.41 (0.28)	0.15
**Mononuclear cell count**				
High (vs normal)	0.01 (0.01)	0.35	0.22 (0.24)	0.35

*EDSS* expanded disability status scale.

*Adjusted for age at sampling, time from sampling, sex region of Sweden, and time on first- and second-line DMTs.

## Discussion

In this nationwide cohort study of cerebrospinal fluid markers in pediatric-onset MS, we found notable differences between PoMS and AoMS, and evidence of a link between OCB-positivity and MS disability early in disease among PoMS. The PoMS cohort had a higher proportion of OCB-positive individuals, and IgG index than the AoMS cohort. While we found no evidence of a relationship between CSF and relapse rate in PoMS, we did find that having OCBs corresponded with higher EDSS levels within 5 years, suggesting that this marker may offer some prognostic utility among persons with pediatric-onset MS.

CSF was first analyzed within the context of inflammatory nervous system diseases in the 1950s and 1960s when both IgG and OCBs were documented in MS patients^18,19^. OCBs represent measurable IgG antibody within the CSF in the absence of corresponding IgG in the serum. Our study suggests that patients with pediatric MS are more likely to be OCB-positive than adult-onset patients. We found higher levels of IgG index, but comparable levels of mononuclear cell counts between PoMS and AoMS.

Previous research has mostly explored PoMS and AoMS separately, but three studies compared the groups^20–22^. Most recently, a single-centre study from the Netherlands reported a high proportion of OCB-positive individuals in the pediatric group (mean age of 15; 91.3% in PoMS vs 87.8% in AoMS)^20^. Two studies from the same clinic in Germany, in which early-onset MS was defined as < 16 years of age, compared PoMS and AoMS^21,22^ and reported no differences in terms of OCB status nor IgG index^21,22^. The PoMS cohort was younger than the current cohort, with a median age of 12.7 years. It’s possible the different findings relate to the age structures of the PoMS cohorts. A 2010 multi-centre study of CSF in PoMS found age-related differences, such that younger children were less likely to exhibit OCBs and elevated IgG indices^23^. The age structure of our cohort precluded us from running a similar analysis, as too few children (< 13 years of age) were included.

Previous efforts to study the relationship between CSF markers and MS disability in PoMS have concentrated on MS risk following an initial demyelinating event^5–7^. Consistently, the evidence has shown that OCB-positive children are much more likely to develop MS than OCB-negative children^5–7^. In the adult MS population, there is a wider literature regarding OCBs and disability, summarized in a 2013 meta-analysis^4^. The pooled analysis of four studies with EDSS disability milestones as the outcome, found an OR of 1.96 (95% CI 1.31–2.94) comparing OCB-positive to OCB-negative individuals^4^. While our study suggests that OCBs may also be associated with worse MS disability, the number of OCB-negative patients was quite low, therefore this analysis should be replicated on larger sample sizes if possible.

Prior research has suggested a relationship between high IgG levels and MS progression, but the focus has largely been on adult cases^24–26^. A single study of children with MS found no evidence of a relationship between elevated IgG index and time to second event^23^, similar to our findings.

Mononuclear cells were elevated in a high proportion of the PoMS cohort, but they were not significantly different from the AoMS, nor were they related to relapse rate or disability.

A strength of our study was the inclusion of incident cases, the vast majority of whom were treatment-naïve, thereby limiting the potential confounding effect of DMT exposure on CSF levels. The SMSreg is a population-based nationwide registry, containing a large number of pediatric patients, followed prospectively. Not all patients enrolled in the SMSreg have CSF values recorded, and fewer have values available within 3 years of their MS onset. It is possible that these patients differ from persons who did not have an LP early in their disease. This should not have affected the analysis comparing AoMS and PoMS, but our full cohort may not be representative of the wider MS population, including persons who are not examined so early in their disease. As always, the true date of MS onset is challenging to elucidate. While we only included CSF values taken within 3 years of reported MS onset, it is possible that the ‘true’ date of MS onset may have preceded this time. This imprecision is unlikely to have affected one group differentially, and therefore should not have biased our results. Last, we modelled the ordinal EDSS as a continuous variable, and did not have the statistical power to stratify based on relapse status. A difference on the EDSS has a clinically different meaning depending on where on the scale it occurs. The PoMS cohort were largely on the low end of the disability scale, as the analyses were limited to the first 5 years of disease, thereby minimizing this potential imprecision.

This study suggested that PoMS tend to more commonly have CSF evidence for intrathecal IgG production than AoMS, including OCBs. Further, we provide evidence for a relationship between incident OCB status and subsequent disability, suggesting that it could play a role in the prognostication of MS in children. Whether active lesions are a component of this relationship in pediatric patients, as observed among adults^27^, remains to be seen. Future studies should aim to incorporate MRI data to better understand these dynamics.

## Data Availability

Data related to the current article are available from Jan Hillert, Karolinska Institutet. To share data from the Swedish MS Registry, a data transfer agreement needs to be completed between Karolinska Institutet and the institution requesting data access. This is in accordance with the data protection legislation in Europe (GDPR [General Data Protection Regulation]). Persons interested in obtaining access to the data should contact Jan Hillert (jan.hillert@ki.se).
